# Temperature-Dependent Non-linear Resistive Switching Characteristics and Mechanism Using a New W/WO_3_/WO_x_/W Structure

**DOI:** 10.1186/s11671-016-1602-7

**Published:** 2016-09-07

**Authors:** Somsubhra Chakrabarti, Subhranu Samanta, Siddheswar Maikap, Sheikh Ziaur Rahaman, Hsin-Ming Cheng

**Affiliations:** 1 Thin Film Nano Tech. Lab., Department of Electronic Engineering, Chang Gung University, 259 Wen-Hwa 1st Rd., Kwei-Shan, Tao-Yuan, 333 Taiwan; 2 Electronic and Opto-electronic Research Laboratories (EOL), Industrial Technology Research Institute (ITRI), Hsinchu, 195 Taiwan; 3 Material and Chemical Research Laboratories (MRL), Industrial Technology Research Institute (ITRI), Hsinchu, 195 Taiwan

**Keywords:** WO_3_ switching material, Temperature, F-N tunneling, Barrier height, Simulation

## Abstract

Post-metal annealing temperature-dependent forming-free resistive switching memory characteristics, Fowler-Nordheim (F-N) tunneling at low resistance state, and after reset using a new W/WO_3_/WO_x_/W structure have been investigated for the first time. Transmission electron microscope image shows a polycrystalline WO_3_/WO_x_ layer in a device with a size of 150 × 150 nm^2^. The composition of WO_3_/WO_x_ is confirmed by X-ray photo-electron spectroscopy. Non-linear bipolar resistive switching characteristics have been simulated using space-charge limited current (SCLC) conduction at low voltage, F-N tunneling at higher voltage regions, and hopping conduction during reset, which is well fitted with experimental current-voltage characteristics. The barrier height at the WO_x_/W interface for the devices annealed at 500 °C is lower than those of the as-deposited and annealed at 400 °C (0.63 vs. 1.03 eV). An oxygen-vacant conducting filament with a diameter of ~34 nm is formed/ruptured into the WO_3_/WO_x_ bilayer owing to oxygen ion migration under external bias as well as barrier height changes for high-resistance to low-resistance states. In addition, the switching mechanism including the easy method has been explored through the current-voltage simulation. The devices annealed at 500 °C have a lower operation voltage, lower barrier height, and higher non-linearity factor, which are beneficial for selector-less crossbar memory arrays.

## Background

Recently, resistive random access memory (RRAM) has become a promising candidate to replace three-dimensional FLASH for crossbar applications at a low cost owing to its simple structure, low power consumption, and high-speed operation [1–4]. Although different switching materials such as Ta_2_O_5_ [5–7], HfO_2_ [8, 9], TiO_2_ [10–12], and Al_2_O_3_ [13–15] have been reported, however, only a few studies have been reported on WO_3_ material [16, 17]. WO_3_ has an acceptable energy gap of 3.25 eV [18] and Gibbs free energy of approximately −529 kJ/mol at 300 K [19]. Chien et al. [16] reported that the Frenkel effect modified the space-charge limited current (SCLC) in a W/WO_x_/TiN structure. Biju et al. [17] reported Schottky emission in low field and Poole-Frankel in high field in a Pt/WO_3_/W structure. Although different structures have been reported to amplify the RRAM characteristics, its temperature-dependent non-linear switching characteristics and mechanism are still unclear [20]. In this regard, the current transport mechanism is one of the key factors in understanding the resistive switching behavior. Many authors have proposed different structures in the current conduction mechanism [21–23]. The barrier height in between the switching material and the electrode can control the interfacial-type bipolar characteristics [5, 21]. On the other hand, non-linear resistive switching characteristics are useful for reducing the sneak path in crossbar architecture, which can be solved using a complementary structure [7, 24] or selector [25]. If the RRAM device shows non-linearity without a selector, then the above issue can be solved in an easy way. Although many structures with different transport mechanism have been reported, a simple W/WO_3_/WO_x_/W RRAM device in the same material has not been reported yet. Non-linear forming-free bipolar resistive switching characteristics using a simple W/WO_3_/WO_x_/W structure are observed for as-deposited, 400 °C, and 500 °C annealed devices. A polycrystalline WO_3_/WO_x_ layer is confirmed by both high-resolution transmission electron microscope (HRTEM) images and X-ray photo-electron spectroscope (XPS) spectra. Temperature-dependent SCLC characteristics at low voltage and Fowler-Nordheim (F-N) tunneling at high voltage for both low-resistance state (LRS) and high-resistance state (HRS) are observed, even after reset. The switching mechanism is explained by oxygen-vacant conducting filament (CF) formation/rupture into the WO_3_/WO_x_ bilayer, and a new method of current-voltage (I-V) simulation is explored. Compared to other memory devices, the devices annealed at 500 °C have higher non-linearity factor, lower operation voltage, and lower barrier heights.

## Methods

**Fig. 1:**
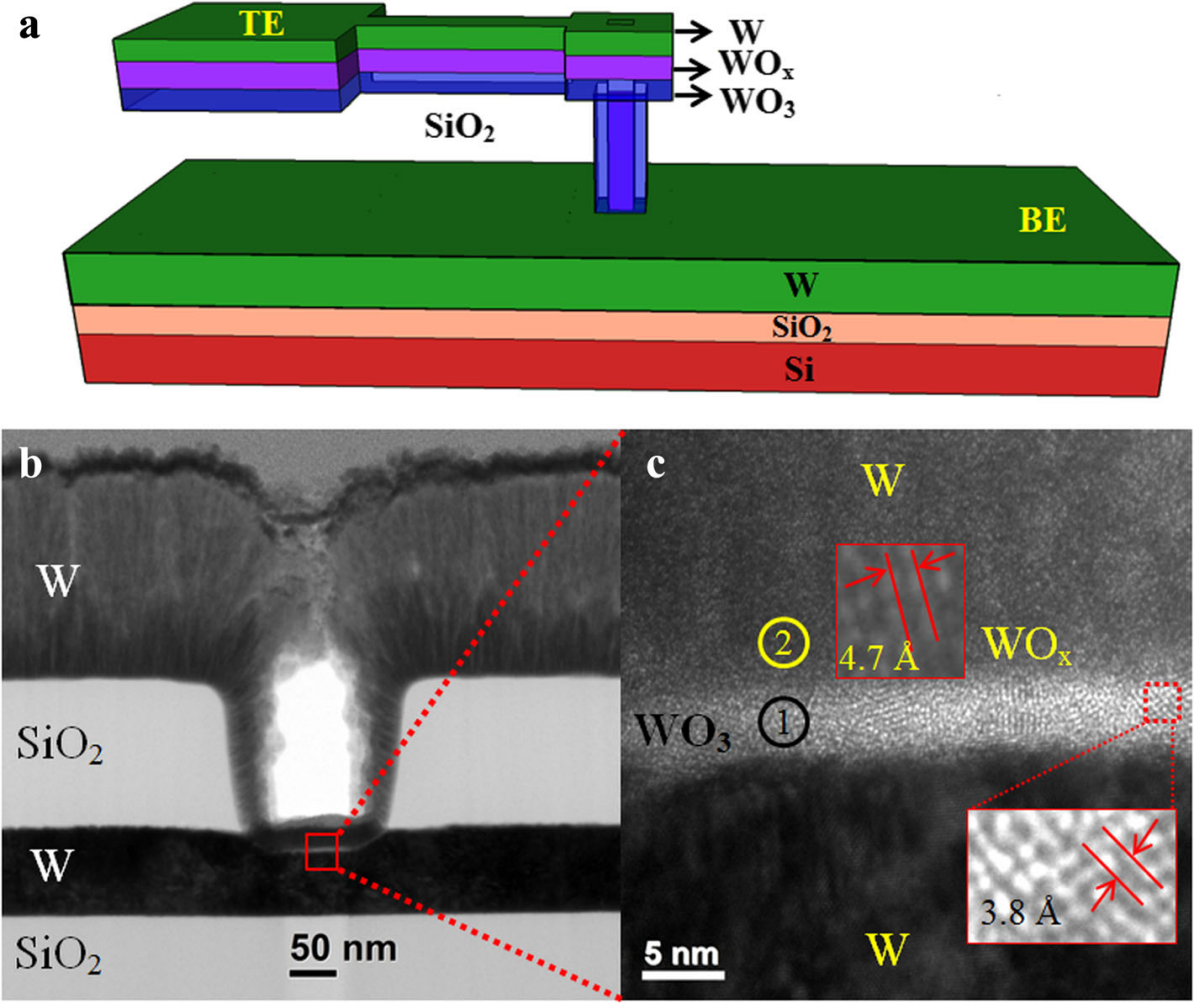
**a** Schematic view of a W/WO_3_/WO_x_/W resistive switching memory device. **b** TEM image shows 150 × 150 nm^2^ devices. **c** HRTEM image confirms the WO_3_/WO_x_ layer. The crystalline WO_3_ and WO_x_ layers with d-spacing are shown inset

First, a Si wafer was cleaned by the standard Radio Corporation of America (RCA) process. Then, a 200-nm-thick SiO_2_ was grown by a thermal oxidation method. A 200-nm-thick tungsten (W) as a bottom electrode (BE) was deposited on the SiO_2_/Si substrate. Then, a SiO_2_ layer with a thickness of approximately 150 nm was deposited by physical vapor deposition method for via-hole patterns. A small via hole with a size of 150 × 150 nm^2^ was formed by a standard photo-lithography process. Then, the WO_3_ layer was deposited by rf sputtering. After that, a WO_x_ layer was deposited, and lastly, W top electrode (TE) was deposited using the same rf sputtering system. The pressure of the sputtering chamber was kept at 10 mTorr during deposition, and the deposition power was 100 W. The flow rate of argon (Ar) gas was 25 sccm during deposition of W TE. By controlling the oxygen (O_2_) flow rate with Ar flow, the WO_3_ layer with a thickness of 4 nm on the BE and the WO_x_ layer with a thickness of 5 nm on the WO_3_ layer were deposited. For the WO_3_ layer, 70 % oxygen is used whereas 30 % oxygen is used for the WO_x_ layer. Finally, a lift-off process was performed to obtain the RRAM devices. These devices were post-metal annealed (PMA) at 400 °C (S2) and 500 °C (S3) for 10 min in ambient N_2_. These annealed devices were compared with the as-deposited one (S1). A schematic view of a RRAM device is shown in Fig. 1a. Memory characteristics were measured by an Agilent 4156C semiconductor parameter analyzer. The sweep voltage was applied on the TE, whereas the BE was grounded during the measurement.

## Results and Discussion

**Fig. 2:**
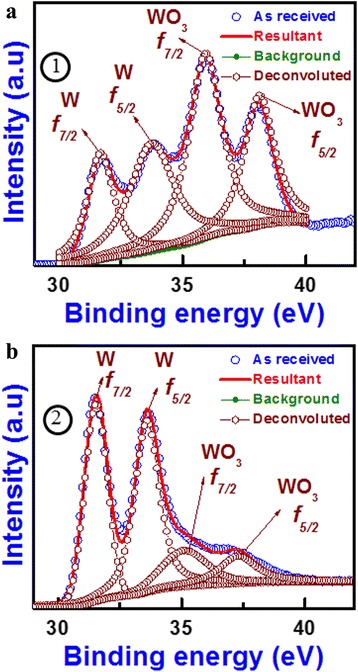
XPS of two areas marked (*1*) and (*2*) in the HRTEM image of Fig. 1c. **a** Region (*1*) shows the presence of WO_3_. **b** Region (*2*) shows the presence of metallic W or WO_x_

Figure 1b shows a TEM image of a RRAM device with a via-hole size of 150 × 150 nm^2^. A WO_3_ switching layer of S1 device with a thickness of an approximately 4-nm layer is shown on the W BE (Fig. 1c). The oxygen-deficient layer, i.e., WO_x_, with a thickness of approximately 5 nm was deposited. Due to the similar material of the WO_x_/W TE, an interface was not observed. The WO_3_/WO_x_ layer was polycrystalline. The polycrystalline grain size will be increased with annealing temperature. Ottaviano et al. [26] reported that the crystallite size of 5-nm-thick WO_3_ changes from 26 to 35 nm due to annealing from 350 to 500 °C. The polycrystalline WO_3_ layer had a d-spacing value of 3.8 Å, which was similar to the reported value of 3.835 Å for the (002) WO_3_ layer [27]. The measured value of d-spacing of WO_x_ was 4.7 Å, which was the same to the reported value of WO_x_ (4.7 Å, [28]). The presence of the WO_3_ and WO_x_ layers was also confirmed by XPS analysis (Fig. 2). Two positions marked (1) and (2) were leveled on the HRTEM image in Fig. 1c, which were obtained from the XPS depth profile of the W TE/WO_x_/WO_3_/W BE sample. By etching layer by layer from the sample surface, the XP spectra were measured. The binding energy peaks centered at 31.6 and 33.8 eV corresponded to the W *f*_7/2_ and W *f*_5/2_, respectively, whereas the peaks centered at 35.9 and 38.1 eV corresponded to the WO_3_*f*_7/2_ and WO_3_*f*_5/2_ core-level electrons, respectively. Those peaks were also confirmed by Kawasaki et al. [29]. It was observed that the WO_3_ intensity at the marked region (1) was stronger than that of the peak at the marked region (2). The atomic percentages of WO_3_ and W were found to be 57.33 and 42.66 % at the marked region (1), respectively, whereas those values were 23.52 and 76.43 % at the marked region (2), respectively. Therefore, marked region (1) was an oxygen-rich layer, i.e., the WO_3_ layer, whereas marked region (2) was an oxygen-deficient layer, i.e., the WO_x_ layer. The resistive switching characteristics of WO_3_/WO_x_ bilayer have been explained below.

**Fig. 3:**
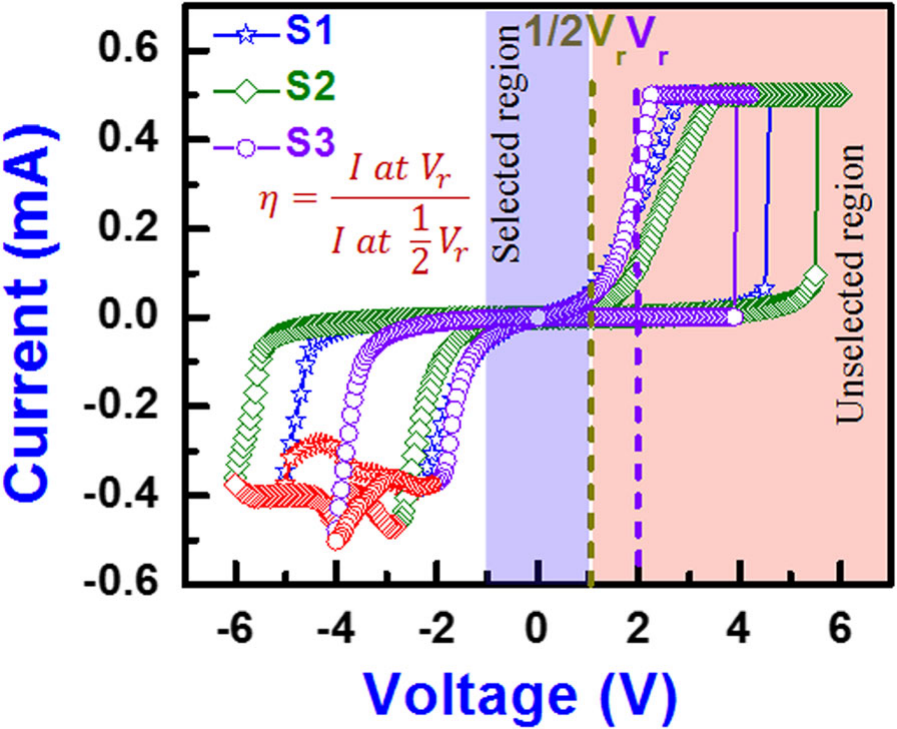
Bipolar resistive switching characteristics of the S1, S2, and S3 devices. The voltage sweep direction is followed: 0 → +Ve → 0 → −Ve → 0 V

**Fig. 4:**
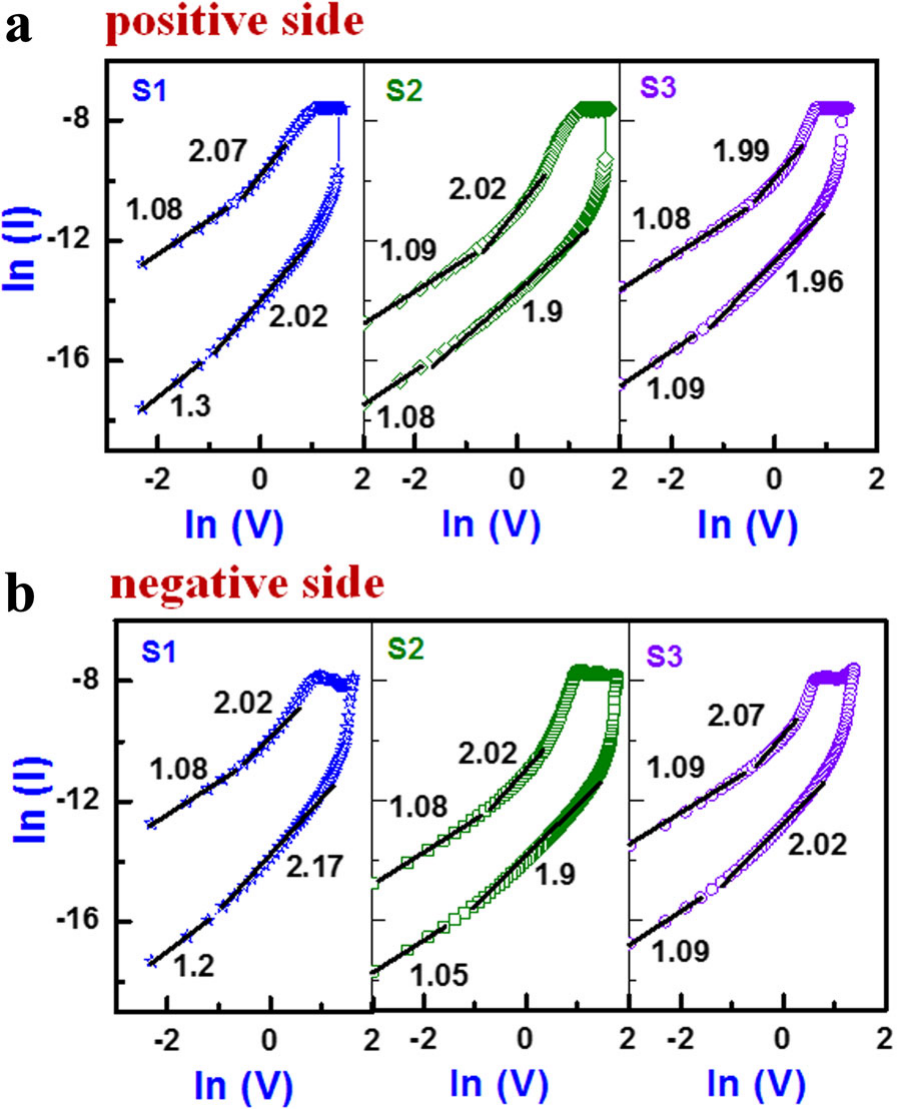
The ln(I)–ln(V) SCLC fitting for LRS and HRS in **a** low positive bias region and **b** low negative bias region

**Fig. 5:**
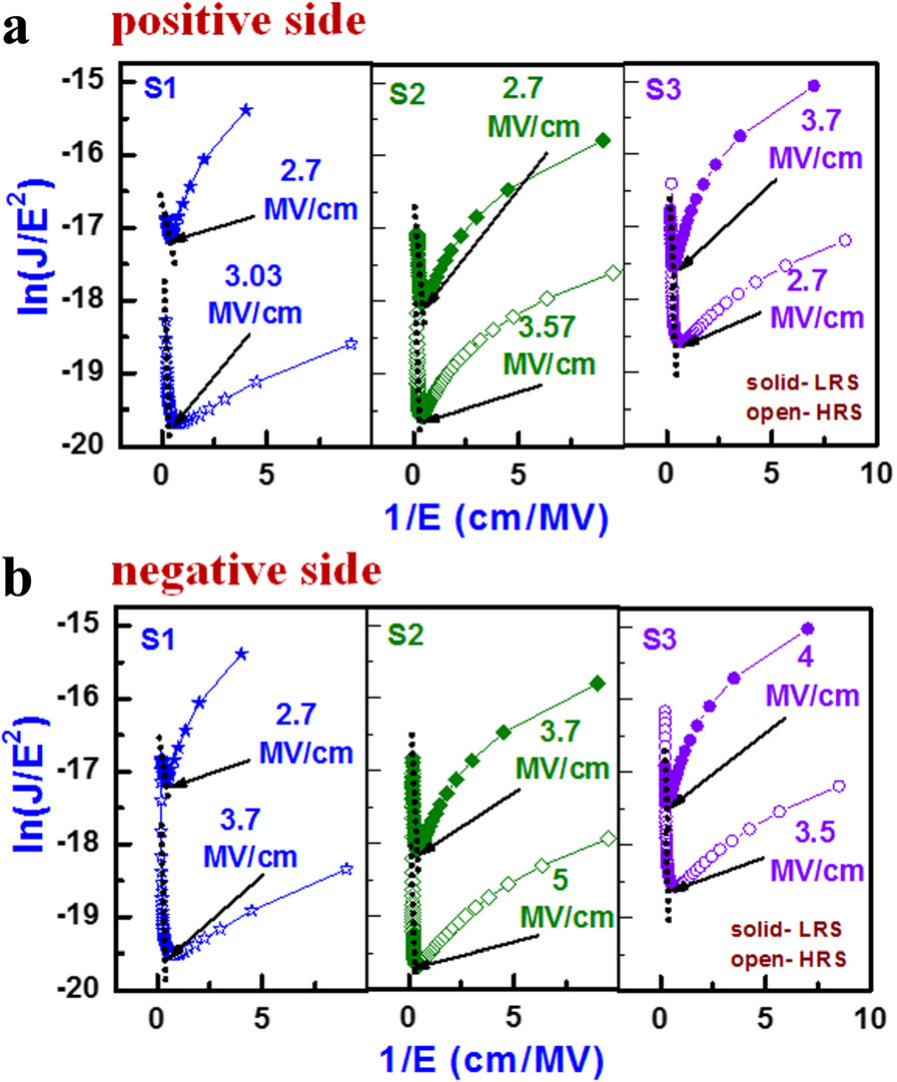
The F-N fitting **a** on the +ve side at set and **b** on the –ve side after reset

Figure 3 shows the I-V characteristics of the S1, S2, and S3 devices under a current compliance (CC) of 500 μA. The voltages of the S1, S2, and S3 devices were set at 4.5, 5.5, and 3.6 V, respectively, and the reset voltages were −2.5, −2.9, and −2.35 V, respectively. These devices were forming free, i.e., the first cycle (on pristine device) is almost similar to the next cycles [30]. During set, the oxygen ions were migrated from the WO_3_ layer by breaking W-O bonds to the WO_x_/W interface and the oxygen-vacancy CF is formed. The device reached to LRS. During reset, oxygen ions were migrated from the WO_x_/W interface to the WO_3_ layer as well as the CF is oxidized and the device reached to HRS. The SCLC [31] was observed at the low bias regions for all the devices.

1 $$J=\frac{9{\epsilon}_r{\varepsilon}_0\mu {V}^2}{8{L}^3}$$

where *J* is the current density, *ε*_*r*_ is the relative permittivity of the insulating material, *ε*_0_ (8.85 × 10^−12^ F/m) is the permittivity of free space, *μ* is the electron mobility, and *L* is the thickness of the switching layer. From the above equation, I-V curves in both positive (+ve) and negative (−ve) bias regions were plotted in ln(I) vs. ln(V) scale (Fig. 4). The SCLC fittings consist of an ohmic region (*I α V*) with slope values from 1.05 to 1.3 and Child’s law region (*I α V*^2^) with slope values from 1.9 to 2.17 for both HRS and LRS. The slope value of the S1 devices is slightly higher (1.3) than unity, but the S2 and S3 devices have close to unity. The reason behind this is the number of defects was decreased after port-metal annealing treatment. Therefore, the S1 devices followed the trap-charge controlled (TC) SCLC whereas the S2 and S3 devices followed SCLC at low bias regions in both HRS and LRS. At the higher bias region of the HRS and LRS, the F-N tunneling equation [31, 32] is below:

2 $$J=\frac{q^3{E}^2}{8\pi hq{\phi}_B}\ \exp \left\{-\frac{8\pi {\left(2q{m}^{*}\right)}^{\frac{1}{2}}}{3 hE}{\phi}_B^{\frac{3}{2}}\right\}$$

where *h* (6.62 × 10^−34^ J s) is Plank’s constant, *q* is electronic charge (1.6 × 10^−19^ C), m* is the effective electron mass, and *E* is the electric field. From F-N tunneling, ln(*J*/*E*^2^) was plotted as a function of 1/*E*. Figure 5a, b shows the F-N tunneling fitting at the +ve and −ve regions for both HRS and LRS. The critical electric field (*E*_*c*_) values at HRS for set were 3.03, 3.57, and 2.7 MV cm^−1^ and those values after reset were 3.7, 5, and 3.5 MV cm^−1^ for the S1, S2, and S3 devices, respectively. It is interesting to note that the F-N tunneling is also observed at LRS because of the oxygen-rich layer formed at the WO_x_/W TE interface, which is reported here for the first time. The *E*_*c*_ values of LRS for the positive region were 2.7, 2.7, and 3.7 MV cm^−1^ and those values before reset were 2.7, 3.5, and 4 MV cm^−1^ for the S1, S2, and S3 devices, respectively. This confirmed that the transport mechanism of both LRS and HRS at the high field regions was dominated by F-N tunneling. A minimum *E*_*c*_ value was found to be 2.7 MV cm^−1^ from all the devices, which was also higher than the reported value of 2.6 MV cm^−1^ [33]. The slope of the F-N fitting curve (Fig. 5) and the value of *Φ*_*B*_ can be calculated by using the equation below:

3 $${\phi}_B = {\left(\frac{3h}{8\pi}\right)}^{2/3}\frac{S^{2/3}}{{\left(2q{m}^{*}\right)}^{1/3}}$$

where *S* is the slope of the fitted line (black dotted lines). The *Φ*_*B*_ values at HRS for the +ve and –ve sides were 0.25/0.56, 0.31/1.03, and 0.28/0.63 eV, while those values at LRS were 0.11/0.10, 0.21/0.28, and 0.25/0.29 eV for the S1, S2, and S3 devices, respectively. All devices showed lower *Φ*_*B*_ in the positive bias region than the negative bias region owing to a higher work function of oxidized W (or WO_x_) than the pure W metal (4.91 eV [34] vs. 4.6 eV [19]). The barrier height (*Φ*_*B*_) values of electrons in HRS for the S2 devices were higher than those of the S1 and S3 devices. This is because of the annealing out of defects from the switching layer at 400 °C. At an annealing temperature of 500 °C, both *Φ*_*B*_ values for the S3 devices were the lower either because of N_2_ incorporated into the WO_x_ layer or the reduction of oxygen and inter-diffusion of W into the WO_3_ layer [35, 36], which can also help lower the operation voltage to ±4 V (Fig. 3). The S3 devices had the benefit of a higher non-linearity factor (*η*), which will help reduce the sneak paths for crossbar array applications [24]. The *η* is defined as *η* = (*I* at *V*_*r*_)/(*I* at 1/2 *V*_*r*_). The values of *η* for the S1, S2, and S3 devices were found to be 5.2, 8.6, and 8.8, respectively. Therefore, we can define the −1 V to 1 V region as the unselected region and the higher voltage region as the selected region, as shown in Fig. 3. This non-linearity resulted from the presence of the WO_3_/WO_x_ bilayer concept in the W/WO_3_/WO_x_/W simple structure. In addition, the S3 devices showed stable data retention of >10^3^ s at a high read voltage of 0.5 V (not shown here). However, CF formation/rupture into the WO_3_/WO_x_ bilayer needs to be explored further, which is discussed below.

**Fig. 6:**
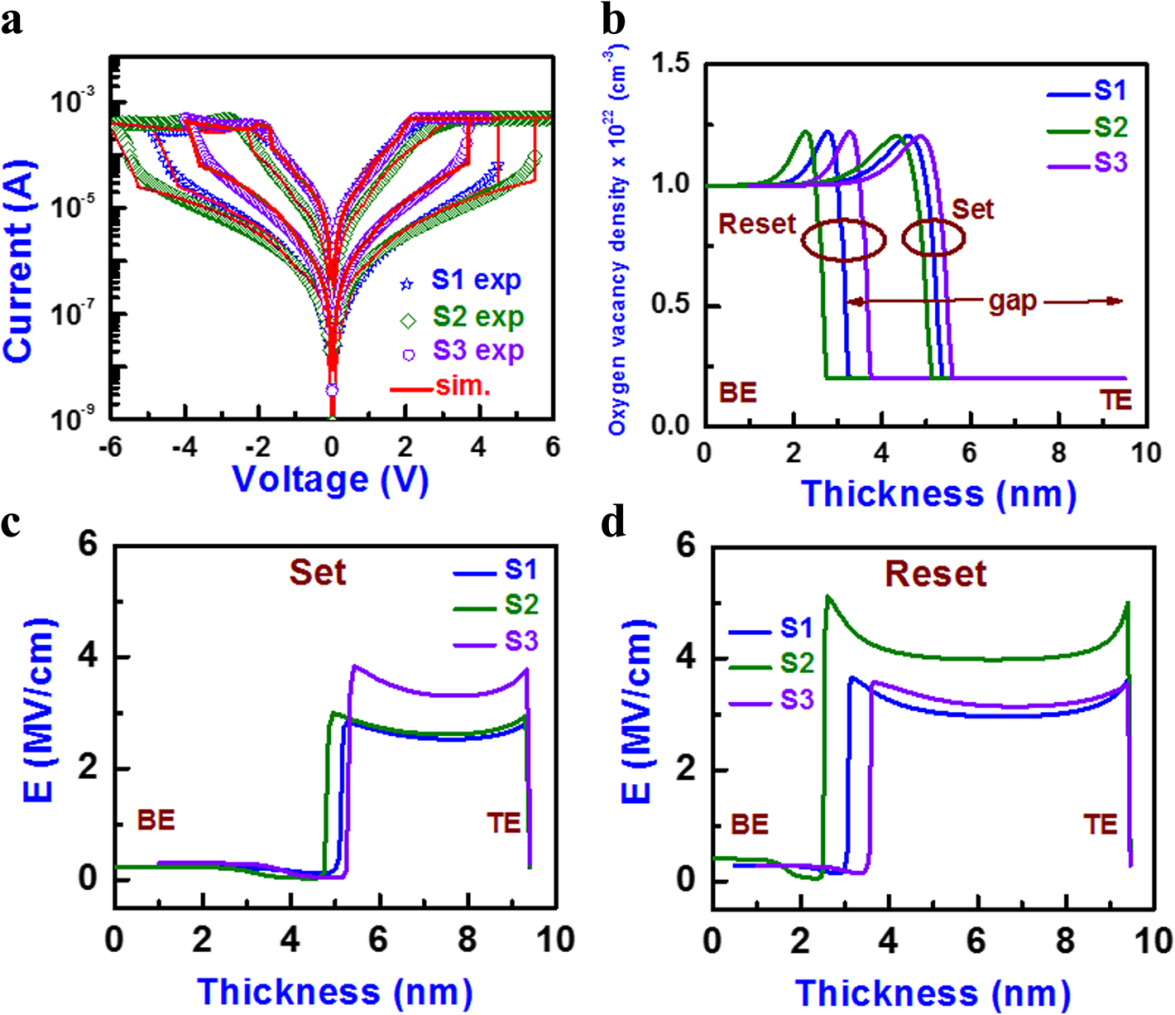
**a** Experimental and simulated I-V in the log scale for the S1, S2, and S3 devices. **b** Oxygen-vacancy density (*n*_*D*_) profiles show a different gap in the set and reset. Corresponding electric field distribution along the CF after **c** set and **d** reset

**Fig. 7:**
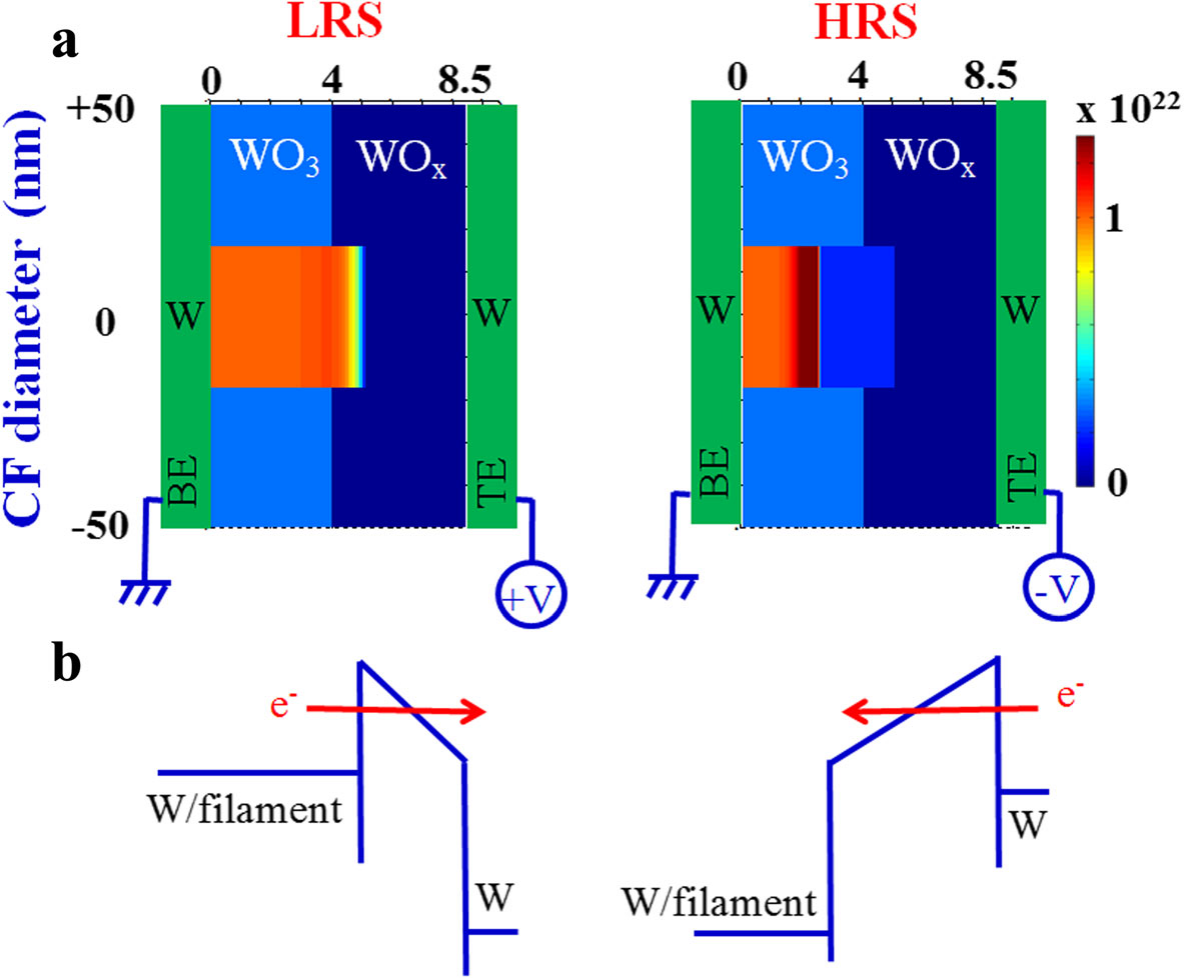
**a** Color map of the oxygen-vacancy filament of the S3 device at both LRS and HRS along with **b** corresponding energy band diagram

The oxygen ion migration under external bias, oxygen-rich layer formation at the WO_x_/W TE interface during set, and larger dissolution gap during reset show the resistive switching characteristics. The transport characteristics are controlled by SCLC at the low bias region and F-N tunneling at higher bias regions for all the devices. Due to oxygen-rich layer formation at LRS, the F-N tunneling is observed, and after reset at the maximum value of negative voltage (−5, −6, and −4 V for the S1, S2, and S3 devices, respectively), the electrons had enough energy to F-N tunnel through the dissolution gap. By using Eqs. (1) and (2) of SCLC and F-N tunneling and using above parameters, the I-V characteristics except reset regions were simulated using MATLAB as a simulation tool. Well-fitted I-V with experimental curve for all devices is shown in Fig. 7a. The input value of *ε*_*r*_ was considered as 5 [37]. The *μ* value through the WO_3_ layer was considered approximately 10^−2^ cm^2^ V^−1^ s^−1^, which is close to the reported value of 5 × 10^−2^ cm^2^ V^−1^ s^−1^ [38]. The *Φ*_*B*_ values obtained from Eq. (2) were considered as those were. The value of effective mass was taken as 0.7 × *m*_0_, which is close to the reported value (in the range of 0.7 × *m*_0_ to 1.2 × *m*_0_ [39]). A similar conduction mechanism was also reported by Kim et al. [40] and Ban and Kim [41] for different structures with switching materials. The reset regions of the S1, S2, and S3 devices were −2.5 to −5 V, −2.8 to −6 V, and −2.1 to −4 V, respectively (i.e., red symbols in Fig. 3). The I-V curves of reset regions were simulated by MATLAB using drift diffusion, current continuity, and Joule heat equations [31]. The oxygen-vacancy flux can be written as the sum of diffusion flux (*J*_*D*_) and drift flux (*J*_*d*_). So total current (*J*_total_) is equal to:

*J*_*D*_ + *J*_*d*_ = − *D*∇*n*_*D*_ + *vn*_*D*_, which is evaluated by:

4 $$\frac{\partial {n}_D}{\partial t}=\nabla .\left(D\nabla {n}_D-v{n}_D\right)$$

5 $$\nabla \sigma \nabla \psi =0$$

6 $$-\nabla {k}_{th}\nabla T=\sigma\ \left|\nabla \psi \right|{}^2$$

where *n*_*D*_ is the *V*_0_ concentration; *t* is the time; *D* [=0.5*a*^2^*f* exp(−*U*_*A*_/*k*_*B*_*T*)] is the diffusivity; *v* [*af* exp(−*U*_*A*_/*k*_*B*_*T*)sinh(*qaE*/*k*_*B*_*T*)] is the drift velocity of oxygen vacancy; *f* is the attempt frequency (10^13^ Hz [42]); *U*_*A*_ is the activation potential of 1 eV, which is similar to the reported values (~1 eV [43]); and *a* is the hopping distance of 0.5 nm, which is similar to our previous reported value (0.56 nm [44]). At zero bias condition, the value of *U*_*A*_ was high. As the voltage was increased, the value of *U*_*A*_ became lower. The electrical conductivity (*σ*) is given by Arrhenius equation: *σ* = *σ*_0_ exp(−*E*_*AC*_/*k*_*B*_*T*), where *σ*_0_ is the pre-exponent constant and *E*_*AC*_ is the activation energy. The *E*_*AC*_ value changes from 0.01 to 0.03 eV, and it is decreasing with increasing value of oxygen-vacancy density (*n*_*D*_), which is similar to the reported value of 0.06 eV [45]. Both the values of *σ*_0_ (WO_3_ = 1.5 × 10^2^ Ω^−1^ m^−1^ [46]; WO_x_ = 7 × 10^2^ Ω^−1^ m^−1^ [47]) and *k*_*th*_ (WO_3_ = 0.2 Wm^−1^ K^−1^; W = 173 Wm^−1^ K^−1^ [19]) varied linearly with the conductivity of WO_3_ to W, and *ψ* was the potential. The value was taken to best fit with the experimental curve. Now we solved Eqs. (4)–(6) simultaneously with the help of MATLAB to obtain the profiles of *n*_*D*_ and *T* with different negative voltages. Consequently, I-V reset curves were obtained. The experimental and simulated I-Vs were given in Fig. 6a. The simulated I-V curves matched quite well with the experimental data. From this simulation, the thickness of the WO_3_ layer was determined to be 4 nm for all structures but the thicknesses of the oxygen-rich WO_x_/W TE interface under set were determined to be 4, 4.5, and 3.5 nm for the S1, S2 and S3 devices, respectively. These thicknesses were also used to calculate *E* in Fig. 5a, b. The cylindrical CF diameter was approximately 34 nm, which is useful for nanoscale non-volatile crossbar array applications. Similar CF diameter of 10–30 nm in a Pt/NiO/Pt structure at a CC of 1 mA was reported by Yun et al. [48]. Yao et al. [49] reported a <1-nm filament diameter in a Au/α-C/SiO_x_/α-C/Au structure with operation current of ~50 μA. Song et al. [50] reported about a 70-nm filament diameter in a Pt/TiO_2_/Pt structure with a CC of 10 mA. Waser and Aono [51] reported an ~12-μm-diameter filament in a Cr-doped SrTiO_3_ single crystal cell with 5-mA current. Celano et al. [13] reported about a 28-nm CF diameter using a Cu/Al_2_O_3_/TiN structure at a CC of 10 μA. Yazdanparast et al. [52] reported the 70-nm CF diameter using a Au/Cu_2_O_3_/Au structure at a CC of 10 mA. According to our previous report [3], the CF diameter is approximately 70 nm in a Cu/GeO_x_/W structure at a CC of >1 mA. A larger diameter of 2 μm using a Pt/CuO/Pt structure was reported by Yasuhara et al. [53]. In addition, the variation of oxygen-vacancy density profiles (*n*_*D*_) with thickness for both the set and reset for all devices are given in Fig. 6b. The value of *n*_*D*_ at the CF is 1 × 10^22^ cm^−3^, and the CF was assumed to be broken if the concentration was below 0.5 × 10^22^ cm^−3^. There is an oxygen-rich layer at the WO_x_/W TE interface under set. The dissolution gap in reset for the devices showed that the device annealed at 500 °C had the smallest gap among the three devices, which was responsible for the lowest set/reset voltage and *Φ*_*B*_ value. Figure 6c shows the *E* (=d*ψ*/d*x*) distributions for the S1, S2, and S3 devices after set (or at LRS). After maximum reset voltages of −5, −6, and −4 V for the S1, S2, and S3 devices, respectively, the *E* distribution along the CF is shown in Fig. 6d. By solving Eqs. (4) and (5) for *ψ* and *n*_*D*_, the *E* profiles were obtained. According to the *E* values at LRS along the CF and after reset (Fig. 5), this shows F-N tunneling (>2.7 MV cm^−1^). Typical color maps of *n*_*D*_ for the S3 devices during set and reset are shown in Fig. 7. The oxygen-rich layer at the WO_x_/W TE interface with a thickness of 3.5 nm was observed at LRS, and the dissolution gap in the CF ruptured region was approximately 7.5 nm. Basically, oxygen ion migration under external bias controls the interfacial oxygen-rich layer and dissolution gap as well as the lower and higher barrier heights which lead to LRS and HRS switching, as shown in energy band diagram under bias (Fig. 7). Comparing all devices, the devices annealing at 500 °C showed higher non-linearity factor with lower operation voltage, and stable data retention at a high read voltage of 0.5 V, which will have the potential for nanoscale non-volatile memory applications. In addition, the I-V switching characteristics using transport and hopping conductions have been simulated using a new and simple concept, which will also help to analyze other resistive switching memory devices in future.

## Conclusions

In conclusion, post-metal annealing effects on the forming-free resistive switching behavior of the W/WO_3_/WO_x_/W structure were observed, especially F-N tunneling at LRS and after reset was observed for the first time. The WO_3_/WO_x_ layer was confirmed by TEM and XPS. The RRAM devices annealed at 500 °C had a lower operation voltage, thinner WO_x_/W TE interface, lower barrier height, and stable data retention. A simulation based on SCLC conduction in the low field, F-N tunneling in the high field for both HRS and LRS, and oxygen-vacant CF with a diameter of ~34 nm was developed for all non-linear I-V switching characteristics, which will be very useful to understand the switching mechanism for other RRAM structures and for selector-less nanoscale crossbar architectures.
